# Vitamin D supplementation for improving children with bone mineral density: A protocol for systematic review and meta-analysis

**DOI:** 10.1097/MD.0000000000023475

**Published:** 2020-12-24

**Authors:** Chengcheng Yuan, Chunyan Qu, Weigang Ji

**Affiliations:** Affiliated Maternity and Child Health Care Hospital of Nantong University, Nantong, China.

**Keywords:** bone mineral density, osteoporosis, protocol, vitamin D supplementation

## Abstract

**Background:**

Osteoporosis is usually one of the less perceived complications of chronic illness among children. Previous studies have shown that vitamin D supplementation may be valuable to bone density, especially among children with a deficiency of vitamin D. Yet, the results often remain inconsistent. Therefore, the present study investigates the clinical therapeutic effects of vitamin D supplementation to enhance children with bone mineral density.

**Methods:**

We will search the randomised controlled experiment literature of vitamin D supplementation for bone mineral density, focusing on children, in 3 distinct English databases (EMBASE, MEDLINE via PubMed, and Cochrane Library) and 2 specific Chinese databases (China National Knowledge Infrastructure (CNKI) and WanFang databases). Additionally, we intend to explore the Clinical Trials.gov, reference lists of identified publication and the grey literature. Accordingly, we will use 2 independent authors to screen the literature, extract data, and research quality assessment. We will carry out all statistical analyses using RevMan 5.3 software.

**Results:**

We will systematically evaluate the clinical therapeutic effects of vitamin D supplementation to enhance children with bone mineral density.

**Conclusion:**

The present study will summarise the currently published pieces of evidence of vitamin D supplementation for bone mineral density in children to further comprehend its promotion and application.

**Ethics and dissemination:**

The present study is a systematic review and meta-analysis founded upon existing or published studies; therefore, ethical approval is not applicable.

**OSF registration number:**

October 24, 2020. osf.io/7vtey. (https://osf.io/7vtey/).

## 1 Introduction

Osteoporosis is a systemic bone disease distinguished by low bone mass, weakening of micro-architectures of bone tissue, which leads to a reduction in bone strength and increases bone fragility or instability and consequently increases the risk of bone fracture.^[1]^ Although often regarded as older peoples disease, osteoporosis has increasingly been identified among children. One of the main determinants of the lifetime risks of the condition is the scale of peak bone strength accomplished by early maturity into old age.^[2]^ In essence, the significant elements of peak bone mass include genetic factors and numerous environmental factors, which are critical to accomplishing the full genetic potential, including nutrition and physical activity.^[3]^ Vitamin D supplementation of children deficient in vitamin D is one of the most valued options to examine for the advancement of peak bone mass.

Previous studies have evaluated bone mass among patients with eating disorders, confirming a link between low bone mineral density and conditions characterised by nutritional deprivation and altered body composition.^[4]^ Subsequently, many studies have indicated that vitamin D can precisely influence bone mass accrual, thus can contribute to controlling calcium-phosphorus metabolism and implicitly stimulate the growth of muscle tissue.^[5–7]^ Serum 25-hydroxyvitamin D levels are also connected to some significant bone density and bone quality parameters among adolescents.^[8]^ In particular, deficiency of vitamin D can be best detected by measuring serum 25-hydroxyvitamin D.^[9]^ An increasing body of evidence have shown that low vitamin D levels in children are widespread enough to regard considerable public health and global issue. However, the efficacy of vitamin D supplementation to enhance children with bone mineral density remains inconclusive. Therefore, this paper aims to examine the clinical therapeutic impacts of vitamin D supplementation to improve children with bone mineral density.

## 2 Methods

### 2.1 Study registration

The procedure is registered with the Open Science Framework (OSF, http://osf.io/), and the registration DOI number for the present study is 10.17605/OSF.IO/7VTEY. Also, the study report is compliant with the guidelines of the Preferred Reporting Items for Systematic Reviews and Meta-Analysis Protocol (PRISMA-P) Statement.

### 2.2 Study inclusion and exclusion criteria

#### 2.2.1 Inclusion criteria

Randomised controlled trials (RCTs) of the clinical therapeutic effects of vitamin D supplementation to improve children with bone mineral density are all included. For the study, language restrictions are English or Chinese.

#### 2.2.2 Exclusion criteria

Non-RCTs, animal studies, case report, repeated publications, and letters will be excluded.

### 2.3 Types of participants

Further, the investigation will comprise of participants with no co-existent medical conditions and any treatments leading to osteoporosis aged below 18 years. We will exclude participants with any treatments causing osteoporosis; studies exclusively performed in neonates aged below one month.

### 2.4 Types of interventions and comparisons

We will plan to include any RCTs of treatment with vitamin D supplementation, different from placebo, no intervention, or other interventions.

### 2.5 Types of outcome measures

The types of outcome measures were areal or volumetric bone mineral density or bone mineral content. Also, the measurement sites will entail lumbar spine, femoral neck, total body, distal forearm, proximal forearm, and total hip.

### 2.6 Search methods

#### 2.6.1 Electronic searches

We will search the randomised controlled experiment literature of vitamin D supplementation for bone mineral density in children, taking into account the 3 English databases (EMBASE, MEDLINE via PubMed, and Cochrane Library) and 2 Chinese databases (China National Knowledge Infrastructure (CNKI), and WanFang databases). The study will further include all qualified reports published from their inception to October 6, 2020. Table 1 provides the search strategy for MEDLINE. The design was customised as a suitable approach for other databases.

**Table 1:** Search strategy for the MEDLINE.

Number	Search terms
#1	MeSH descriptor: [Bone Density] explode all trees
#2	“Bone mineral Density^∗^”:ti,ab,kw
#3	“Bone mineral Density”:ti,ab,kw
#4	“Density, Bone”:ti,ab,kw
#5	“Density, Bone Mineral”:ti,ab,kw
#6	“osteoporosis^∗^”:ti,ab,kw
#7	#1 or #2 or #3 or #4 or #5 or #6
#8	MeSH descriptor: [Vitamin D] explode all trees
#9	“Vitamin D”:ti,ab,kw
#10	“vitamin D supplementation”:ti,ab,kw
#11	#8 or #9 or #10
#12	MeSH descriptor: [randomised controlled trials] explode all trees
#13	“randomized controlled trial”:ti,ab,kw
#14	“controlled clinical trial”:ti,ab,kw
#15	(“randomised^∗^” or“randomized^∗^” or “placebo^∗^” or “randomly” or “trial^∗^”):ti,ab,kw
#16	#12 or #13 or #14 or #15
#17	#7 and #11 and #16

#### 2.6.2 Other resources

Additionally, we intend to examine ClinicalTrials.gov, reference lists of well-known publication, as well as grey literature.

### 2.7 Data collection and analysis

#### 2.7.1 Studies selection

**Figure 1:**
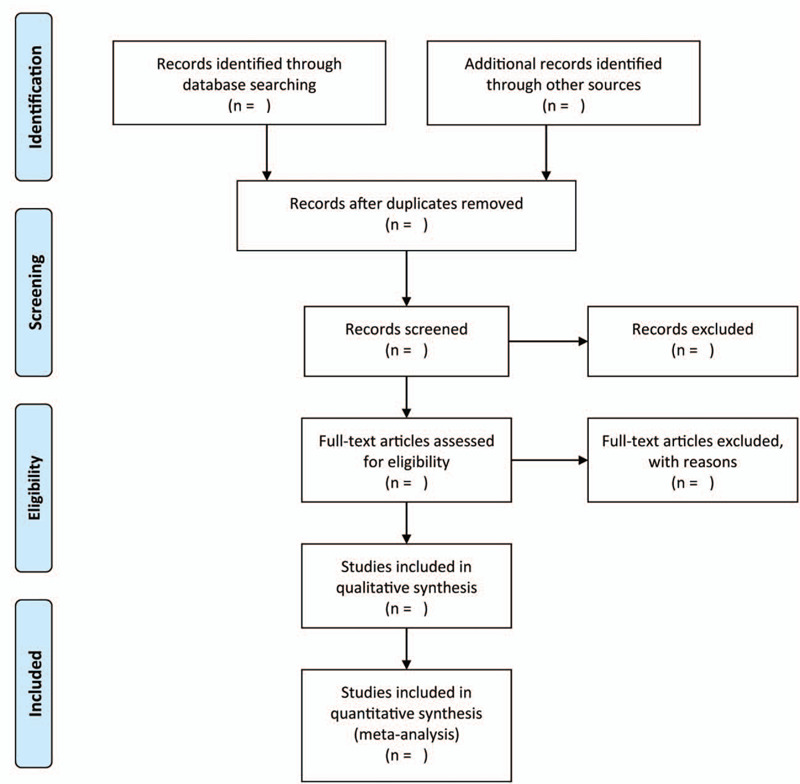
Flowchart of study selection.

We will use 2 independent authors to evaluate the literature identified. EndNote X9 will be used to remove duplicated publications. Then, we will read the full texts of relevant works of literature to select further those that satisfy the inclusion criteria. In case of any disagreements in the process, they shall be addressed through deliberation and an agreement will be reached. Figure 1 displays the research flow chart.

#### 2.7.2 Data extraction and management

Two independent authors will further obtain data into the pre-designed Microsoft Excel 2019 software table. The table will include first author, publication year, gender, mean age, duration, number of cases, height, weight, dietary calcium intake, baseline serum vitamin D, vitamin intake, calcium supplement use, body mass index, and physical activity.

#### 2.7.3 Assessment of risk bias

Furthermore, the 2 independent authors will examine the risk of bias by applying the Cochrane Collaboration's “Risk of bias” tool.^[10]^ Based on the 6 domains (random sequence generation, allocation concealment, blinding, incomplete data, selective outcome reports, and other bias), the authors will classify the risk of bias into “high risk”, “low risk”, or “unclear”. Further discrepancies will be addressed through deliberation to reach an agreement.

#### 2.7.4 Measures of treatment effect

The dichotomous outcome data will be presented as a risk ratio (RR) with 95% confidence intervals (CI). We will further demonstrate the continuous outcome data as mean difference (MD) or standardised mean difference (SMD) with 95% CI.

#### 2.7.5 Dealing with missing data

In the case of indistinct or misplaced data, we will get in touch with the respective authors to supply additional information where necessary.

#### 2.7.6 Assessment of heterogeneity

We will determine the heterogeneitys assessment using by Chi^2^ statistic and *I*^2^ test. When the value of *I*^2^ > 50% or *P* value <.1, it means that there is significant heterogeneity. However, when the value of *I*^2^ < 50% or *P* value <.1, there is no evident statistic heterogeneity. We will plan to use the random-effects model to pool the data where significant heterogeneity will be detected.^[11]^

#### 2.7.7 Assessment of reporting biases

The funnel plot and Egger test will be utilised to evaluate reporting biases if selected studies are sufficient.^[12,13]^

#### 2.7.8 Sensitivity analysis

We will use sensitivity analysis to evaluate each outcome indicator to establish the stability of outcome indicators.

## 3 Discussion

Although published pieces of evidence have reported that vitamin D supplementation can enhance bone mineral density among children, the findings are still inconclusive. The present investigation aims to examine the clinical therapeutic effects of vitamin D supplementation to enhance children with bone mineral density. The results of the present study will provide a foundation for vitamin D supplementation to improve children with bone mineral density and suggest treatment options of osteoporosis patients, which could benefit more patients.

## Author contributions

Conceptualization: Chengcheng Yuan and Chunyan Qu; Data curation and formal analysis: Chengcheng Yuan, Chunyan Qu, and Weigang Ji; Investigation: Chengcheng Yuan and Weigang Ji; Methodology: Chunyan Qu; Project administration: Weigang Ji; Resources: Weigang Ji; Software: Chengcheng Yuan; Supervision: Weigang Ji; Validation: Chengcheng Yuan and Chunyan Qu; Visualization: Chengcheng Yuan; Writing - original draft: Chengcheng Yuan; Writing - review and editing: Chengcheng Yuan and Chunyan Qu.

**Conceptualization:** ChengCheng Yuan, chunyan qu.

**Data curation:** chunyan qu.

**Funding acquisition:** ChengCheng Yuan, chunyan qu.

**Investigation:** ChengCheng Yuan.

**Methodology:** ChengCheng Yuan, Weigang ji.

**Project administration:** Weigang ji.

**Resources:** ChengCheng Yuan, Weigang ji.

**Software:** ChengCheng Yuan, Weigang ji.

**Supervision:** Weigang ji.

**Writing – original draft:** ChengCheng Yuan, chunyan qu.

**Writing – review & editing:** ChengCheng Yuan, chunyan qu.
